# Ultrafast Dynamics of Plasmon-Mediated Charge Transfer in Ag@CeO_2_ Studied by Free Electron Laser Time-Resolved X-ray Absorption Spectroscopy

**DOI:** 10.1021/acs.nanolett.0c04547

**Published:** 2021-02-11

**Authors:** Jacopo Stefano Pelli Cresi, Emiliano Principi, Eleonora Spurio, Daniele Catone, Patrick O’Keeffe, Stefano Turchini, Stefania Benedetti, Avinash Vikatakavi, Sergio D’Addato, Riccardo Mincigrucci, Laura Foglia, Gabor Kurdi, Ivaylo P. Nikolov, Giovanni De Ninno, Claudio Masciovecchio, Stefano Nannarone, Jagadesh Kopula Kesavan, Federico Boscherini, Paola Luches

**Affiliations:** † Elettra-Sincrotrone Trieste S.C.p.A., Strada Statale 14 km 163.5 in Area Science Park, 34012 Basovizza, Trieste, Italy; ‡ Dipartimento FIM, Università degli Studi di Modena e Reggio Emilia, Via Campi 213/a, 41125 Modena, Italy; § Istituto Nanoscienze, Consiglio Nazionale delle Ricerche,Via G. Campi 213/a, 41125 Modena, Italy; ∥ Division of Ultrafast Processes in Materials (FLASHit), Istituto di Struttura della Materia (ISM-CNR), Area della Ricerca di Roma 2 Tor Vergata, Via del Fosso del Cavaliere 100, 00133 Rome, Italy; ⊥ Division of Ultrafast Processes in Materials (FLASHit), Istituto di Struttura della Materia (ISM-CNR), Area della Ricerca di Roma 1, I-00015 Monterotondo, Scalo, Italy; # Laboratory of Quantum Optics, University of Nova Gorica, Nova Gorica SI-5000, Slovenia; ∇ IOM, CNR, s.s. 14, Km. 163.5 in AREA Science Park, 34149 Basovizza, Trieste, Italy; ○ Dipartimento di Fisica e Astronomia, Alma Mater Studiorum − Università di Bologna, Viale C. Berti Pichat 6/2, 40127 Bologna, Italy

**Keywords:** time-resolved XAS, FEL, plasmonic nanoparticles, CeO_2_, ultrafast charge transfer

## Abstract

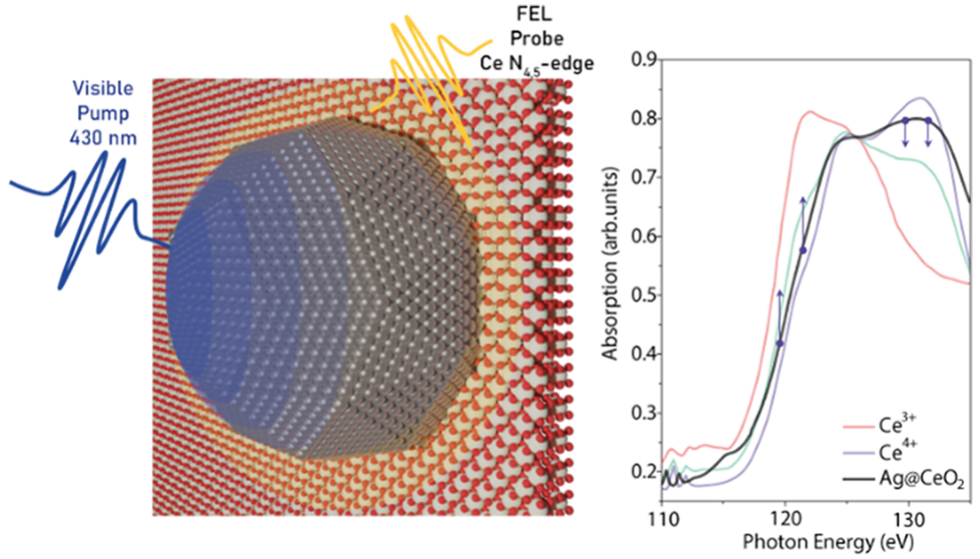

Expanding the activity of wide bandgap semiconductors from the UV into the visible range has become a central goal for their application in green solar photocatalysis. The hybrid plasmonic/semiconductor system, based on silver nanoparticles (Ag NPs) embedded in a film of CeO_2_, is an example of a functional material developed with this aim. In this work, we take advantage of the chemical sensitivity of free electron laser (FEL) time-resolved soft X-ray absorption spectroscopy (TRXAS) to investigate the electron transfer process from the Ag NPs to the CeO_2_ film generated by the NPs plasmonic resonance photoexcitation. Ultrafast changes (<200 fs) of the Ce N_4,5_ absorption edge allowed us to conclude that the excited Ag NPs transfer electrons to the Ce atoms of the CeO_2_ film through a highly efficient electron-based mechanism. These results demonstrate the potential of FEL-based TRXAS measurements for the characterization of energy transfer in novel hybrid plasmonic/semiconductor materials.

In recent years, the use of solar light to drive catalytic reactions has become a potential green alternative to traditional thermally driven heterogeneous catalysis.^1^ Successful implementation of solar-based photocatalysis in applications such as H_2_O splitting and CO_2_ reduction would give an important boost to the renewable energy technologies industry.^2,3^ Transition metal oxides (TMOs) such as TiO_2_, ZnO, or CeO_2_, have very good catalytic properties, but they exhibit rather poor light-induced activity in the visible range as an effect of their wide bandgaps. Several studies have demonstrated that the combination of these materials with plasmonic nanoparticles (NPs) can effectively extend their photoactivity into the visible region of the spectrum.^4−7^ The strong interaction between metal nanostructures and visible light triggers localized surface plasmon resonances (LSPR) accompanied by an energy and/or charge transfer process from the plasmonic material to the neighboring oxide that boosts the TMO reactivity in the visible range. Moreover, the formation of a Schottky barrier at the interface between the metal and the TMO hinders the fast recombination of the injected electrons (holes), increasing their probability of being involved in redox reactions.^5^

The LSPR de-excitation can activate the TMO through three main groups of mechanisms:^8^ (i) electron transfer processes, dominating in the first hundreds of femtoseconds,^9−12^ (ii) photothermal conversion, prevailing on the picosecond time scale when electron–phonon and phonon–phonon scattering become the dominant channels,^13,14^ (iii) photonic enhancement or electric field enhancement,^15^ which can excite the oxide only for photon energies greater than the band gap and prevails for NP with diameters above a few tens of nm. These specific mechanisms have to be understood and optimized to efficiently exploit the energy/charge transfer processes in the various applications involving solar energy conversion, such as photocatalysis or photovoltaics. This represents a challenging task, which can be achieved by studies of the dynamic evolution of the systems after LSPR excitation.

CeO_2_ (cerium oxide or ceria) plays an important role as a TMO catalyst. Its electronic configuration, with a bandgap in the ultraviolet range (3.2–4.0 eV),^16,17^ allows fast and reversible changes between the more stable 4+ oxidation state and the 3+ oxidation state of Ce with one extra electron localized in the Ce 4f levels between the filled valence band and the empty conduction band. This property is associated with the remarkable capability of ceria to store, transport, and release oxygen depending on the environmental conditions. The combination of ceria with metallic NPs can induce modifications of important properties, such as oxygen vacancy formation energy, which may improve the material reactivity, increasing the prospect of using sunlight for environmental and energy applications.^18^ An increase of the sensitivity of Ag NPs/CeO_2_ systems to visible light has been recently highlighted;^19−21^ however, the mechanisms involved in the LSPR-mediated enhancement remain poorly understood. Recent experiments, carried out on a UV-Vis time-resolved facility on the Ag NPs/CeO_2_ system,^4^ revealed a potential electron transfer process from NPs to the oxide occurring on the subpicosecond time scale. In general, ultrafast spectroscopies in the ultraviolet, visible, and infrared range lack chemical sensitivity,^4,22^ thus limiting the interpretation of the results to qualitative considerations.

Time-resolved X-ray absorption spectroscopies (TRXAS) represent a valuable tool to obtain element-specific information on light-triggered ultrafast processes, providing very high sensitivity to the fine details of the electronic structure of the individual elements present in the investigated materials.^23^ Ultrashort pulses (from femtoseconds to hundreds of femtoseconds) of soft X-rays can be generated by both free-electron laser (FEL) and high harmonic generation sources. In recent years, high harmonic sources have demonstrated their potential to provide time-resolved information on processes occurring in functional materials.^24,25^ However, FEL sources are particularly suitable for ultrafast core-level spectroscopies, as they guarantee high pulse intensities in a rather large spectral range. In particular, the FERMI FEL in Trieste (Italy) gives the possibility of finely tuning the photon energy within the 20–300 eV photon energy range, guaranteeing a remarkable spectral stability and nearly transform-limited pulses.^26^ These features, accompanied by an almost jitter-free laser-FEL synchronization,^27^ are ideal for laser pump-X-ray probe single-shot experiments. In this work, we carry out a time-resolved XAS experiment on a light activated Ag NPs/CeO_2_ sample with the aim of exploring the ultrafast changes occurring in the electronic structure of Ce and finally shedding light on the nature of the charge transfer from Ag NPs to CeO_2_ following LSPR excitation.

The Ag NPs/CeO_2_ samples employed in this study were grown at the SESAMo laboratories in Modena on ultrathin (100 nm) parylene-N self-standing foils, as soft X-ray transparent substrates (Figure 1b). Mass-selected Ag NPs, with an average diameter *d* of ∼20 nm (Figure 1a,c), were grown by an inert gas aggregation cluster source based on magnetron sputtering. The NPs were coevaporated with cerium oxide forming a film with embedded NPs, hereafter referred to as Ag@CeO_2_.^4^ This specific configuration is considered better than the CeO_2_-supported Ag NPs scheme, as it maximizes the interface region, where the metal–oxide interaction takes place, and protects the Ag NPs from contamination. A cerium oxide film of the same thickness without Ag NPs was also grown for reference. The quantity of CeO_2_ and silver deposited were estimated to be the product of the evaporation time and the deposition rates of the sources obtained by a quartz microbalance. The samples were characterized by X-ray photoelectron spectroscopy in situ (see Supporting Information) and transferred under inert atmosphere to the experimental setups for optical, XAS, and time-resolved XAS characterization.

**Figure 1:**
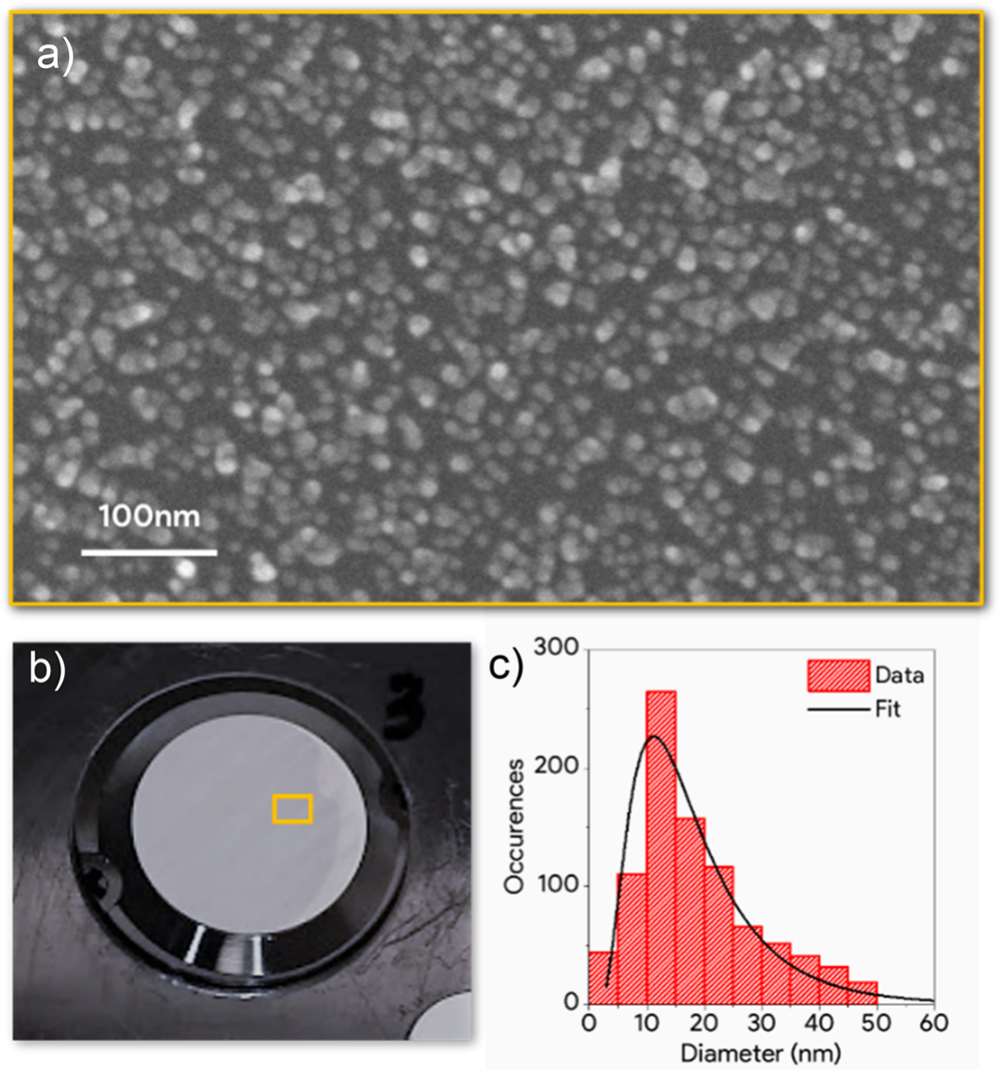
(a) SEM image of a sample made of Ag NPs on a CeO_2_ film grown on a Si substrate. (b) Picture of the Ag@CeO_2_ sample on a parylene substrate. (c) Ag NPs size distribution extracted from (a).

The samples were preliminarily characterized using steady UV–Vis spectrophotometry, to verify the response of the Ag@CeO_2_ system to visible light and to identify the spectral features of Ag NPs and CeO_2_. The spectra, in Figure 2, show that the absorbance of the ceria film without Ag NPs exhibits a peak at 300 nm (blue line). This spectral feature is compatible with excitations from the valence band to empty 4f levels, in agreement with the literature.^16,17^ The Ag@CeO_2_ sample shows broad and intense absorbance peaks centered at 400 and 650 nm, ascribed to the LSPR excitation of Ag NPs. The specific origin of the two peaks was investigated through simulations of the absorbance of Ag NPs in a CeO_2_ matrix, performed using the boundary element method, as implemented in the MNPBEM17 toolbox.^28^ The simulations show that the peaks can be ascribed to different specific configurations of the Ag NPs within the CeO_2_ matrix and to extended plasmon resonances introduced by the proximity of the NPs (details in the Supporting Information).

**Figure 2:**
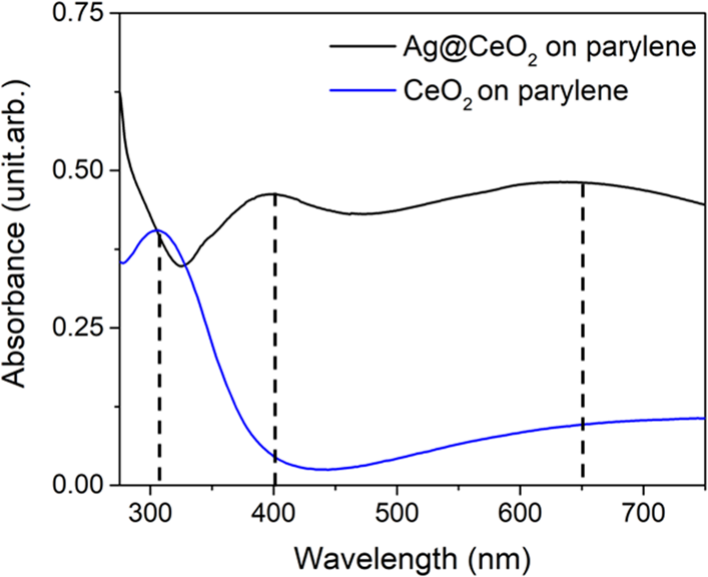
Absorbance spectra of the Ag@CeO_2_ film (black) and of a ceria film (blue).

Stationary XAS spectra of the samples at the Ce N_4,5_ edge have been carried out at the BEAR IOM-CNR synchrotron radiation beamline at Elettra (Trieste, Italy)^29^ for reference. Figure 3 shows the spectra for the CeO_2_ film and the Ag@CeO_2_ sample after pre-edge background subtraction (red and black solid lines, respectively). The spectrum of the CeO_2_ film is compatible with the Ce^4+^ reference spectrum reported in the literature,^30^ shown as a dashed green line in Figure 3. The addition of Ag NPs to the CeO_2_ system induces a red shift of about 1 eV of the Ce absorption edge and a tangible decrease of the white line height. Both effects are compatible with a mild reduction of cerium oxide in the Ag@CeO_2_. A more pronounced reduction of the oxide in the Ag@CeO_2_ sample is expected to lead to a spectral shape more similar to the Ce^3+^ reference spectrum^30^ (dashed blue line). The reference spectrum was acquired on CeCl compound because this compound is more stable in air compared to Ce_2_O_3_, which can easily become oxidized to CeO_2_. The observed reduction in Ag@CeO_2_ results from electron transfer from Ag NPs to the oxide^31,32^ typical of metal NP/oxide systems and it should involve the ceria at the interface. The X-ray photoemission spectroscopy measurements reveal that the surface Ce^3+^ concentration is comparable to that estimated from the small changes in the Ce N_4,5_ edge (see Figure S1, Supporting Information). This is probably caused by the morphology of the sample which induces the presence of defects on the ceria surface.

**Figure 3:**
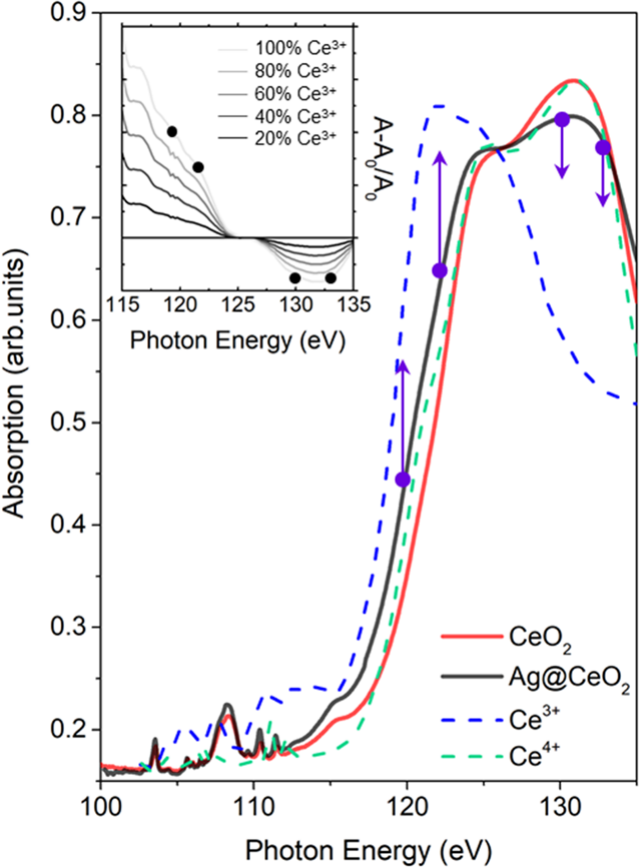
Ce N_4,5_ XAS absorption spectra measured in transmission mode for CeO_2_ (solid red line) and Ag@CeO_2_ (solid black line) samples. The spectra of Ce^4+^ (dashed green line) and Ce^3+^ (dashed blue line) reference samples taken from the literature (30) are also reported. These spectra were normalized to the maximum of the Ce^4+^ spectrum measured in our experiment. The inset reports the relative variation of absorption during the reduction of Ce (Ce^4+^ → Ce^3+^) estimated from the literature spectra. Purple points, and the black points in the inset, indicate the selected FEL energies used to probe the variations of absorption.

The TRXAS measurements have been performed at the EIS-TIMEX end-station^33^ of the FERMI FEL (Trieste, Italy) operating in single-shot laser pump-FEL probe mode (Figure 4) on the Ag@CeO_2_ sample at selected energies across the Ce N_4,5_-edge. An ultrashort laser pulse at 430 nm is used as a pump to selectively excite the Ag NPs LSPR. Indeed, the optical absorption of the CeO_2_ matrix is negligible at the pump energy if compared to the Ag NPs one (see Figure 2). Moreover, the interband transitions in Ag are characterized by weak cross sections at this photon energy. Four specific FEL photon energies (119, 122, 130 and 133 eV), marked by the purple arrows in Figure 3, were chosen to maximize the sensitivity to possible changes in the electronic structure in Ce ions driven by ultrafast reduction of CeO_2_. The FEL and the laser pump beam diameters on the sample were 80 and 100 μm, respectively. The pump pulse duration was estimated to be about 200 fs, while the average FEL probe pulse duration was around 100 fs. The instrument response function (IRF) of the setup is thus dominated by the pump laser duration. The laser pump fluence was set to about 34 mJ cm^–2^.

**Figure 4:**
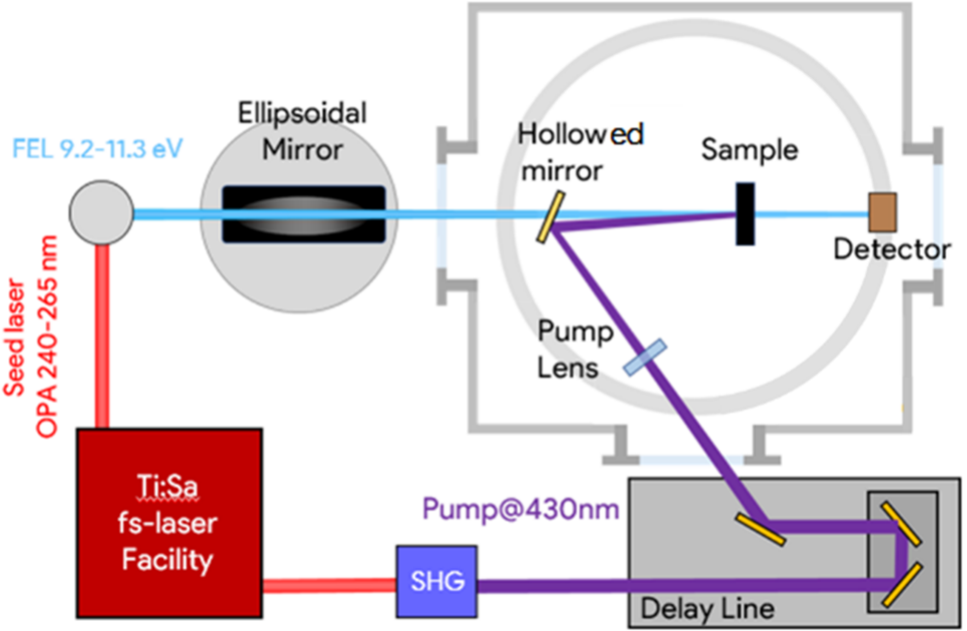
EIS-TIMEX end-station setup for pump–probe XAS measurements in transmission geometry. Small angles between pump and FEL are achieved using a holey steering mirror positioned in the FEL beam path. Synchronization between the laser pump and FEL probe is nearly jitter-free being both the pulses generated by the same Ti:Sa oscillator.

The TRXAS measurements were performed in the transmission mode by rastering the sample in the plane perpendicular to the beam and illuminating fresh regions of the sample at each pump shot. The transmission of the unperturbed sample in every fresh position was measured by exposing the sample to a sequence of probe pulses prior to pump exposure. The delay time between the pump and the probe pulses was scanned with steps of 0.1 ps within a range of about 1 ps from their overlap time. Repeated single-shot measurements at each delay time in different positions have been carried out on the sample, thus obtaining good counting statistics for each chosen photon energy. In order to account for possible nonuniformities of the sample, we have excluded the uppermost and the lowermost 5% of the distribution of the measured changes in transmission for each energy and delay. Time zero was calibrated using Si_3_N_4_ as usually performed for FEL/Vis pump cross-correlation.^34^ It is possible that slight variations in the zero delay from run to run (e.g., changing the photon energy of the FEL) can result in a variation of the delay by up to 100 fs. Figure 5 reports the relative variation of the absorption, as a function of the pump–probe delay time, at the selected FEL photon energies across the Ce N_4,5_-edge. In particular, the X-ray absorption coefficient after excitation exhibits a pronounced increase at 119 and 122 eV of about 10% (Figure 5a,b) and a decrease at 130 and 133 eV of about 5% (Figure 5c,d).

**Figure 5:**
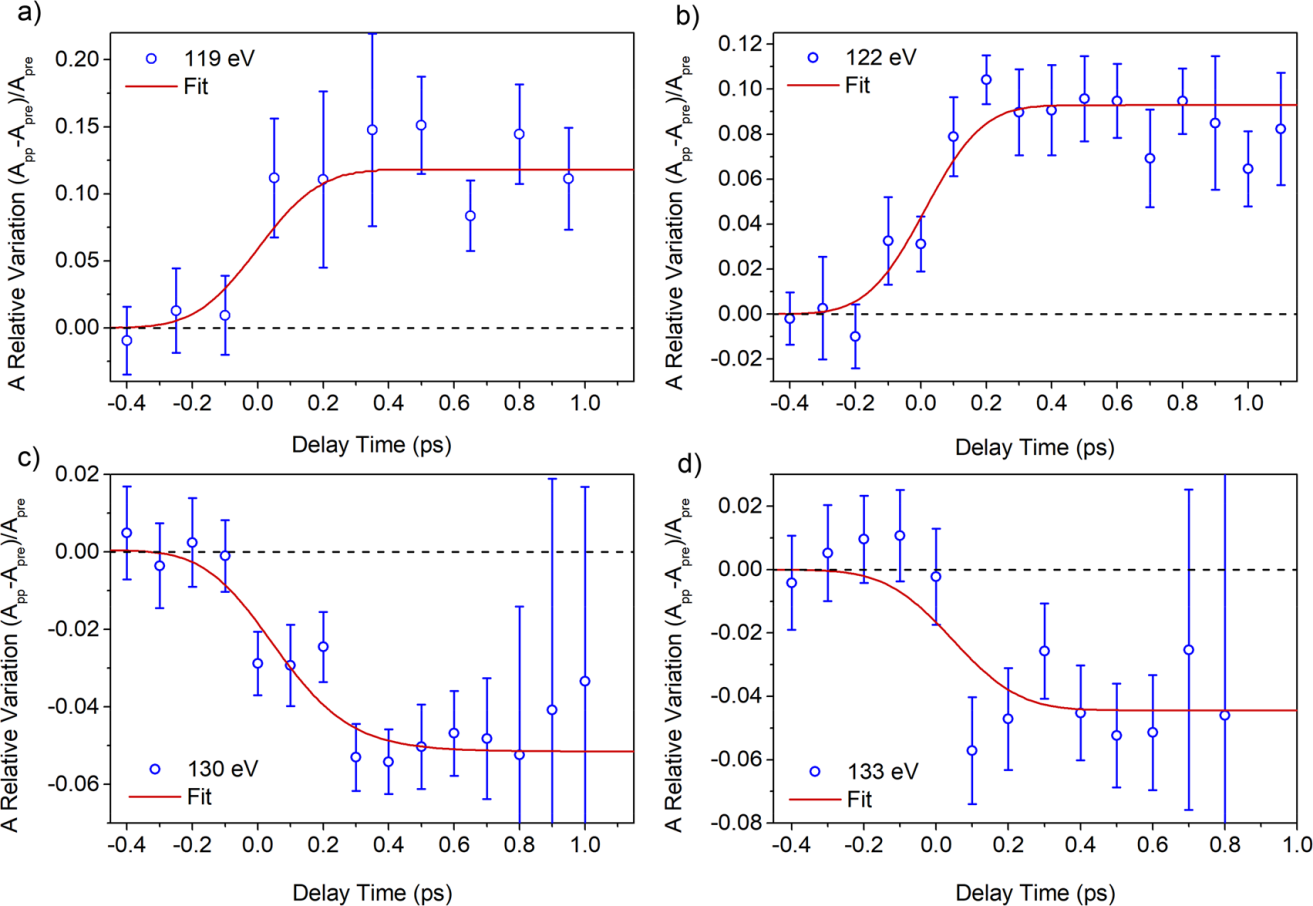
Relative variation of absorption as a function of pump–probe delay time and corresponding fit (red curve) at (a) 119 eV, (b) 122 eV, (c) 130 eV, and (d) 133 eV.

The changes in the X-ray absorption occur within the first few hundred femtoseconds and persist at least up to about 1 ps delay time between the pump and the probe. The inset of Figure 3 shows the expected relative absorption changes of Ce N_45_ XAS spectra if the electronic configuration of Ce is progressively modified by adding electrons in the 4f levels (calculated using the reference spectra and assuming different concentrations of Ce^3+^). A pronounced increase of absorption below 125 eV and a moderate decrease above 125 eV are expected with the increasing concentration of Ce^3+^ ions, in agreement with TRXAS findings. Therefore, the absorption change shown in Figure 5 appears to be compatible with an ultrafast reduction of part of the Ce ions surrounding the Ag NPs driven by a LSPR-mediated electron injection in the Ce 4f localized states. Possible contributions of multiphoton absorption processes directly in the CeO_2_ material were excluded by exciting a sample of CeO_2_ without the Ag NPs with a pump fluence similar to that used on the Ag@CeO_2_ sample. The resulting variation in probe absorption (for a single FEL energy at 130 eV), reported in Figure S2 of the Supporting Information, does not show any evident dynamics.

The data in Figure 5 were fit using a kinetic profile (obtained by the product of an exponential functions and a step function) convoluted with an IRF of Gaussian shape with fwhm of 200 fs, compatible with the width of the pump laser (details in the Supporting Information). The results of the fit show that the rise time of the signal is compatible with the pump pulse duration. This evidence confirms that the charge transfer process is faster than a typical thermal process (electron–phonon scattering), which requires typical rising times of 1–10 ps.^35,36^ The negligible decay of the transient XAS signal at long delay times up to 1 ps is consistent with the long-lived excited state that we observed in previous experiments using a visible probe^4^ and suggests that the charge injected in 4f states does not recombine rapidly. The amplitude of the observed absorption variations, estimated by the fit, can be related to the density of electrons transferred to ceria. Using the reference spectra of Ce^3+^ and Ce^4+^ samples,^30^ we related the measured variations in absorption at the different probe energies to the fraction of Ce ions affected by injected electrons. The resulting fraction, estimated to be about 20%, indicates that charge transfer involves the cerium atoms contained in the volume of the first interfacial cerium oxide monolayer (0.312 nm) that surrounds the Ag NPs. A semiquantitative estimation of the efficiency is reported in the Supporting Information. This estimate supports the high efficiency for the plasmon-mediated electron transfers that we observed in previous UV–Vis time-resolved experiment^4^ and it suggests that by combining Ag NPs with ultrathin oxide shells the plasmon-induced oxide excitation can be maximized.

The experiment described here represents the first application of FEL-based time-resolved XAS to a hybrid plasmonic NPs/oxide material. The TRXAS measurements reveal that the electronic structure of the Ce atoms undergoes an ultrafast change following photoexcitation of the LSPR in the Ag NPs. The sign and the amplitude of the observed variations at the different energies and their ultrafast nature demonstrate that the decay of the LSPR in the Ag NPs involves electron transfer processes, which is the dominant process below 1 ps. This result demonstrates clearly that the FEL-based characterizations of novel hybrid functional materials are tangible and very promising. For this reason we believe that this experiment will pave the way to the study of kinetics and mechanisms of charge transfer in photocatalytic material through FEL-based experiments.

## Supporting Information Available

XPS characterization of the Ag@CeO_2_ and CeO_2_ samples; TRXAS data on CeO_2_; boundary element method simulations of extinction spectra of Ag NPs in different configurations; fitting procedure of the dynamics; estimation of the electron injection efficiency (PDF)
