# Supplementary material for: Ultrafast Dynamics of Plasmon-Mediated Charge Transfer in Ag@CeO2 Studied by Free Electron Laser Time-Resolved X-ray Absorption Spectroscopy
Source: Nano Lett. 2021 Feb 11;21(4):1729–34. doi: 10.1021/acs.nanolett.0c04547 (PMC8023697; doi:10.1021/acs.nanolett.0c04547)
Supplement: Supplementary file 1 — nl0c04547_si_001.pdf [file nl0c04547_si_001.pdf]

Supporting Information

Ultrafast Dynamics of Plasmon-Mediated Charge Transfer in Ag@CeO_2 studied by Free Electron Laser time-resolved X-ray Absorption Spectroscopy

Jacopo Stefano Pelli Cresi[1,], Emiliano Principi[1], Eleonora Spurio[2,3], Daniele Catone[4], Patrick O'Keeffe[5], Stefano Turchini[4], Stefania Benedetti[3], Avinash Vikatakavi[2,3], Sergio D'Addato[2,3], Riccardo Mincigrucci[1], Laura Foglia[1], Gabor Kurdi[1], Ivaylo P. Nikolov[1], Giovanni De Ninno[1,6], Claudio Masciovecchio[1], Stefano Nannarone[7], Jagadesh Kopula Kesavan[8], Federico Boscherini[8] and Paola Luches[3]*

[1] Elettra-Sincrotrone Trieste S.C.p.A., Strada Statale 14 km 163.5 in Area Science Park, 34012 Basovizza, Trieste, Italy

[2] Dipartimento FIM, Università degli Studi di Modena e Reggio Emilia, Via Campi 213/a, 41125 Modena, Italy.

[3] Istituto Nanoscienze, Consiglio Nazionale delle Ricerche,Via G. Campi 213/a, 41125 Modena, Italy.

[4] Istituto di Struttura della Materia (ISM-CNR), Division of Ultrafast Processes in Materials (FLASHit), Area della Ricerca di Roma 2 Tor Vergata, Via del Fosso del Cavaliere 100, 00133 Rome, Italy.

[5] Istituto di Struttura della Materia (ISM-CNR), Division of Ultrafast Processes in Materials (FLASHit), Area della Ricerca di Roma 1, I-00015 Monterotondo Scalo, Italy

[6] Laboratory of Quantum Optics, University of Nova Gorica, Nova Gorica, Slovenia

7 IOM,CNR, s.s. 14, Km. 163.5 in AREA Science Park, 34149 Basovizza, Trieste, Italy

[8] Dipartimento di Fisica e Astronomia, Alma Mater Studiorum – Università di Bologna, Viale C. Berti Pichat 6/2, 40127 Bologna, Italy

[*] corresponding author: jacopostefano.pellicresi@elettra.eu

X-ray photoemission spectra

Pure ceria and Ag@CeO_2 were characterized after the deposition on the parylene-N substrate by in situ XPS using Al K_α photons. The Ce^{3+} concentration was estimated by fitting Ce 3d XPS spectra using Ce^{3+} and Ce^{4+} components, as in Skala et al. (*1*). The spectra and their fit are reported in Figure S1, while the resulting concentrations of Ce^{3+} are reported in table S1. The Ce^{3+} concentration is comparable in the two samples within the errors, estimated to be approximately ±3%, and it can be ascribed to defects on the film surface.

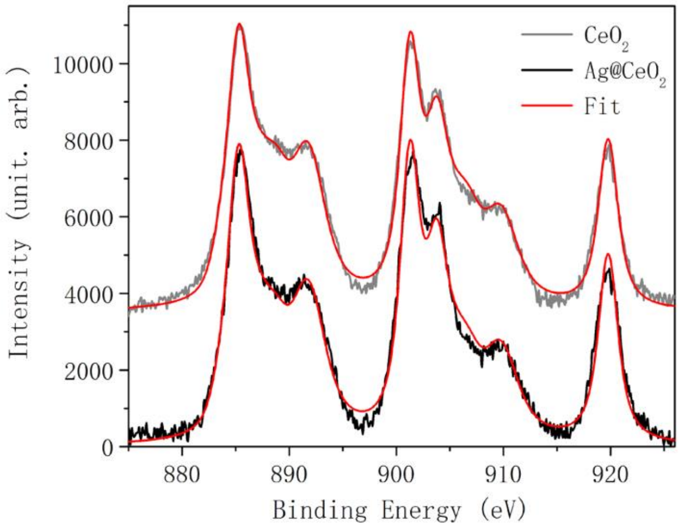

Figure S1 Ce 3d XPS spectra of cerium oxide (grey curve) and of Ag@CeO2 (black curve) grown on parylene. The fitting obtained using Ce^{3+} and Ce^{4+} related components are also shown (red curve).

Sample	Ce^{4+} conc.	Ce^{3+} conc.
CeO_2	82 %	18 %
Ag@CeO_2	86 %	14 %

Table S1 Ce^{3+} surface concentration estimated from the fitting of Ce 3d XPS spectra in the two samples. The uncertainties, limited to ±10%, are intrinsic to the fitting procedure.

TRXAS on a CeO2 film

In order to observe potential multiphotonic absorption effects in CeO_2, a control experiment on a bare cerium oxide film (i.e. without the Ag nanoparticles) was performed under similar conditions. As expected from an almost signal-free measurement, the data are very noisy (they are obtained for about 90 series of shots for single delay), however it can be stated that no significant change in the probe absorption is observed (Figure S2). Therefore, we believe they allow us to exclude a significant contribution of direct multiphoton absorption of the pump within the cerium oxide to the change of the absorption of the soft X-ray probe in the AgNP@CeO_2 system.

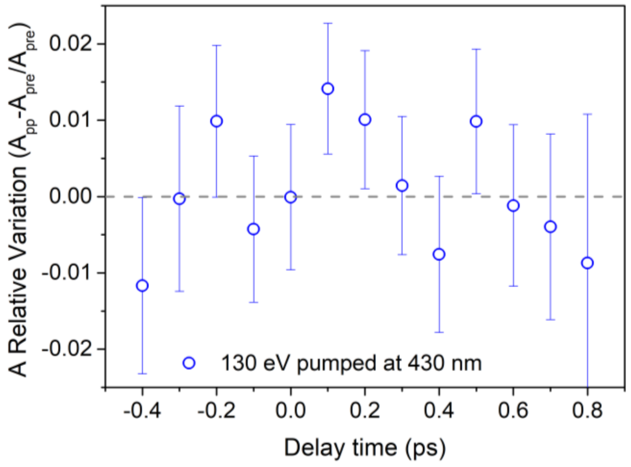

Figure S2 Relative variation of absorption of CeO_2 as a function of pump-probe delay time. The probe energy was 130 eV while the pump wavelength was 430 nm.

Boundary Element Method Simulations

To obtain information on the origin of the plasmonic features in the UV-Vis absorption spectrum, we performed calculations using the MNPBEM, a toolbox developed by Hohenester and coworkers, which uses the boundary element method (BEM) approach developed by Garcia de Abajo and A. Howie (*2*). It permits to calculate the scattering and the extinction coefficients of metal NPs surrounded by a dielectric material. The dielectric constants were extracted from tabulated n and k values taken from the literature (Ag from Palik (*3*), CeO_2 from Kim (*4*) and parylene from (*5*)). We assume a sample geometry composed by spheroids with in-plane size of 20, 10 nm and a height of 20 nm. This geometry, presented in Figure S3, is compatible with the possible agglomeration of the preformed NPs during the deposition and it was already observed in previous studies (*6*).

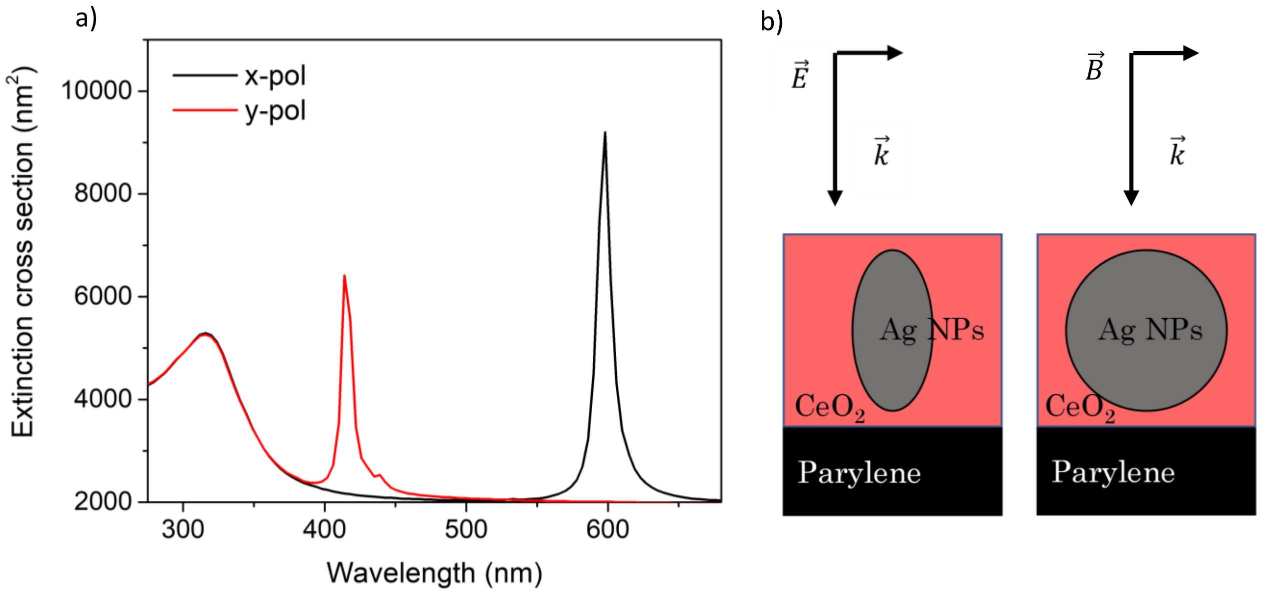

Figure S3 Extinction spectra obtained for an Ag spheroid of 20x10x20 nm (vertical section presented in the sketch) embedded in a layer of cerium oxide of 21 nm on parylene, considering two in-plane orientations of the light polarization, parallel (red line) and perpendicular (black line) to the short axis of the NP.

Figure S3 shows the simplest case: a single Ag NP embedded in CeO_2. The extinction cross section exhibits a strong peak below the 350 nm, compatible with the cerium oxide band-gap absorption. Moreover, two additional peaks at 410 nm and 600 nm are observed if the light polarization is parallel or perpendicular to the shorter in-plane axis of the spheroid, respectively. These spectral features are ascribed to LSPR modes and they are compatible with the experimental spectrum. We also extended

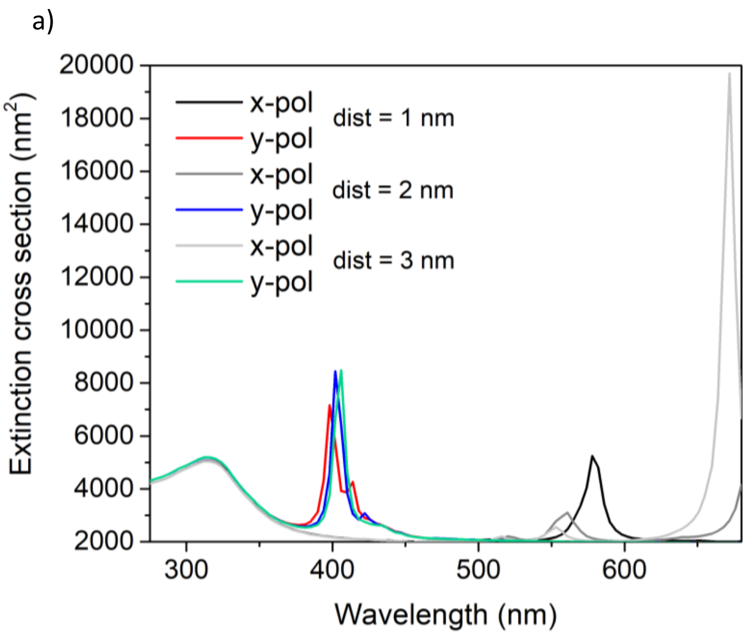

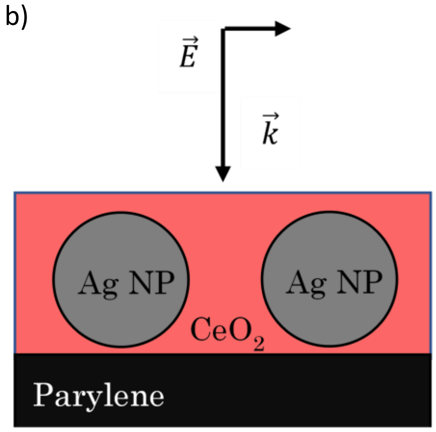

Figure S4 Extinction spectra obtained for two Ag spheroids of 20x10x20 nm (vertical section presented in the sketch) embedded in a layer of cerium oxide of 21 nm on parylene.

the simulation to a couple of NPs with same shape (Figure S4b) to investigate the effect of extended plasmons. We have considered several distances to compare the results. In Figure S4a, we observe 2 main plasmonic features at 400 nm and 585 nm which are compatible with the experimental spectrum.

Dynamics Fitting Procedure

The dynamics were fit using a kinetic profile (obtained by the product of an exponential function and a step function) convoluted with an IRF of Gaussian shape with FWHM of 200 fs ($\sigma \sim 0.15\, ps$), compatible with the width of the pump laser:

$$\Delta A(t) = \int_{-\infty}^{\infty} \frac{1}{\sqrt{2 \cdot \sigma} \cdot \sqrt{\pi}} e^{-\frac{t^2}{\sqrt{2} \cdot \sigma}} \cdot A \cdot \; e^{-\frac{t-c}{\tau_1}} \cdot \theta(t-c) \cdot dt \,,$$

where θ is the Heaviside function (the step function), can offset of the timing zero, A the intensity and τ_1 the characteristic rise time of the kinetic profile.

Estimation of electron injection efficiency

We assume a uniform sample of Ag NPs with size 20x10x20 nm (Figure S5). The NPs form a film with an equivalent thickness of 9.4 nm (measured with a quartz balance). The NPs are embedded in a film of CeO_2 with an equivalent thickness of 11.5 nm, as also measured using a quartz balance. Considering 0.312 nm as the thickness of a monolayer of ceria (in the most stable 111 orientation), the ratio between the volume of interface ceria V_{IF} and the total ceria V_{TOT} can be estimated as:

$$V_{IF} = \frac{4}{3}\pi\big((\,10.312\, nm * 10.312\, nm * 5.312\, nm) - (10\, nm * 10\, nm * 5\, nm)\big) = 272\, nm^3$$

$$V_{TOT} = V_{Ag} * \frac{11.5}{9.4} = \frac{4}{3}\pi(\,10\, nm * 10\, nm * 5\, nm) * \frac{11.5}{9.4} = 2562\, nm^3$$

$$\frac{V_{IF}}{V_{TOT}} = 10.6\,\%$$

In our experiment, we observed a variation of Ce^{3+} edge absorption of 20%. Thus, more ceria than that present at the interface is reduced by the plasmon mediated mechanism in the first 200 fs.

In first approximation, the number of injected electrons is the number of cerium ions contained in 20% of CeO_2 film:

$$n_{el} = \frac{V_{excCe}}{V_{Ce}} = \frac{2562\ nm^3 \cdot 0.2}{0.541^3 nm^3/4} = 12944\ electrons$$

where V_{Ce} is the volume occupied by a cerium atom in the cerium oxide unit cell (4 atoms per unit cell) and V_{excCe} is the volume estimated to be excited by the pump laser (20% of V_{TOT}, the total volume of the ceria film).

The number of absorbed photons by a NPs n_{ph} can be calculated from the static absorbance $A(\lambda_{pump})$ measured at 430 nm, the pump fluence F, the in-plane surface of a NP (s = 10nm · 5nm · π = 157 nm^2), as:

$$n_{ph} = \frac{A(\lambda_{pump}) \cdot F \cdot s}{\hbar\omega_{pump}} \sim 1.6 \cdot 10^5\ photons$$

Thus, an estimate of the injection efficiency η is:

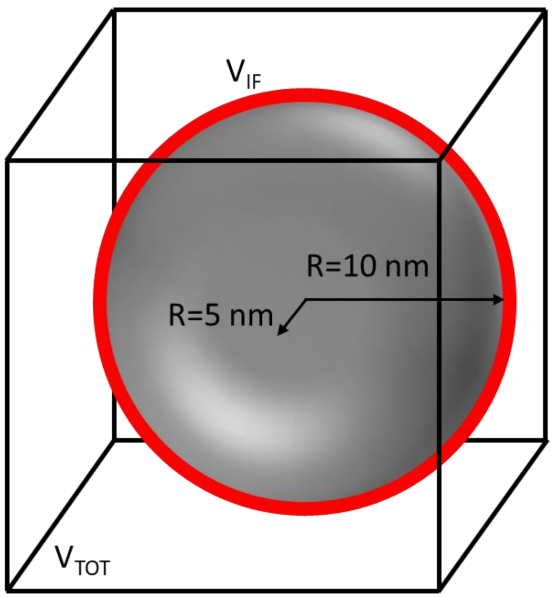

Figure S5 Sketch of a NP with radius of 10x5x10 nm. The red circle represents the volume of CeO_2 at the interface of the NP.

$$\eta = \frac{n_{el}}{n_{ph}} = \frac{12944\ electrons}{1.6 \cdot 10^5\ photons} \sim 8\%$$

BIBLIOGRAPHY

1. T. Skála, F. Šutara, K. C. Prince, V. Matolín, Cerium oxide stoichiometry alteration via Sn deposition: Influence of temperature. *J. Electron Spectrosc. Relat. Phenom.* **169**, 20–25 (2009).

2. F. J. García de Abajo, A. Howie, Retarded field calculation of electron energy loss in inhomogeneous dielectrics. *Phys. Rev. B*. **65**, 115418 (2002).

3. J. Barth, R. Johnson, M. Cardona, E. Palik, *Handbook of optical constants of solids II* (Academic Press, New York, II., 1991).

4. W.-H. Kim, W. J. Maeng, M.-K. Kim, J. Gatineau, H. Kim, Electronic structure of cerium oxide gate dielectric grown by plasma-enhanced atomic layer deposition. *J. Electrochem. Soc.* **158**, 217 (2011).

5. T. E. F. M. Standaert, P. J. Matsuo, X. Li, G. S. Oehrlein, T.-M. Lu, R. Gutmann, C. T. Rosenmayer, J. W. Bartz, J. G. Langan, W. R. Entley, High-density plasma patterning of low dielectric constant polymers: A comparison between polytetrafluoroethylene, parylene-N, and poly(arylene ether). *J. Vac. Sci. Technol. Vac. Surf. Films*. **19**, 435–446 (2001).

6. J. S. P. Cresi, M. C. Spadaro, S. D'Addato, S. Valeri, S. Benedetti, A. D. Bona, D. Catone, L. D. Mario, P. O'Keeffe, A. Paladini, G. Bertoni, P. Luches, Highly efficient plasmon-mediated electron injection into cerium oxide from embedded silver nanoparticles. *Nanoscale*. **11**, 10282–10291 (2019).
